# Stability, Balance, and Physical Variables in Patients with Bilateral Hemophilic Arthropathy of the Ankle versus Their Healthy Peers: A Case–Control Study

**DOI:** 10.3390/life14081051

**Published:** 2024-08-22

**Authors:** Carlos Truque-Díaz, Javier Meroño-Gallut, Cristina Molina-García, Rubén Cuesta-Barriuso, Raúl Pérez-Llanes

**Affiliations:** 1 Faculty of Physiotherapy, Podiatry and Occupational Therapy, Catholic University San Antonio-UCAM, 30107 Murcia, Spain; ctruque@ucam.edu (C.T.-D.); cmolina799@ucam.edu (C.M.-G.); 2 Tú. Bienestar 360°, Physiotherapy and Medical Center, 30730 San Javier, Spain; ajmerono@gmail.com; 3 InHeFis Research Group, Instituto Asturiano de Investigación Sanitaria (ISPA), 33011 Oviedo, Spain; rperez@um.es; 4 Department of Surgery and Medical-Surgical Specialties, University of Oviedo, 33003 Oviedo, Spain; 5 Department of Physiotherapy, University of Murcia, 30100 Murcia, Spain

**Keywords:** hemophilia, balance, range of motion, functionality, strength

## Abstract

(1) Background: The recurrence of hemarthrosis in patients with hemophilia triggers a pathophysiological process of degenerative, progressive, and irreversible joint destruction. This hemophilic arthropathy is characterized by chronic pain, muscle atrophy, loss of mobility, and proprioceptive alterations. As the same joint undergoes repeated hemarthrosis, the function of the mechanical receptors deteriorates, causing a pathophysiological modulation and deterioration of the musculoskeletal system. The objective was to analyze the differences in stability and balance, as well as in ankle dorsal flexion, functionality, and muscle strength, between patients with bilateral hemophilic arthropathy and their healthy peers. (2) Methods: A cross-sectional descriptive case–control study was performed. Twenty-two participants were recruited: 10 adult patients with bilateral hemophilic arthropathy of the knee and ankle and 12 healthy subjects. The variables were balance (Rs Scan pressure platform), ankle dorsiflexion range of motion (Leg Motion), functionality (2-Minute Walk Test), and ankle dorsal strength (dynamometry). (3) Results: Statistically significant differences (*p* < 0.05) were found in the balance without visual support in the Max-Y variable (MD = 2.83; CI95%: 0.33;5.33; Effect size (d) = 0.67), ankle dorsiflexion (MD = 16.00; CI95%: 14.30; 20.0; d = 7.46), and strength of the ankle flexor muscles (MD = 128.50; CI95%: 92.50; 153.60; d = 2.76). (4) Conclusions: Ankle range of motion in dorsal flexion, functionality, and muscle strength in dorsal flexion is poorer in patients with bilateral lower limb hemophilic arthropathy than in their healthy peers. Patients with bilateral hemophilic ankle arthropathy have statistically poorer stability and balance without visual support than their healthy peers.

## 1. Introduction

Hemophilia is a rare disease in which one of the clotting factors is decreased. The two missing factors are FVIII (hemophilia A) and FIX (hemophilia B). The severity phenotypes of the disease, depending on the percentage of missing factors in the blood, are mild (>5%), moderate (1–5%), and severe (0–1%) [1]. The incidence of hemophilia A is approximately 1:5000 live births, while that of hemophilia B is approximately 1:20,000 [2].

The clinical manifestation of hemophilia includes hemarthrosis. This bleeding, which can develop spontaneously or in the event of trauma, mainly affects the elbows, knees, and ankles [1]. The recurrence of hemarthrosis promotes the accumulation of hemosiderin in the synovial membrane, causing chronic synovial swelling and stimulating bone and cartilage degeneration [3]. The process of joint degeneration, hemophilic arthropathy, is characterized by chronic pain, muscle atrophy, loss of mobility, and proprioceptive alterations. These limitations reduce the amount of activities, social participation, and quality of life of patients with hemophilia [4].

The ankle is the joint in which patients with hemophilic arthropathy report the most pain [5]. The main alterations of ankle arthropathy are joint deformity, decreased range of motion, mainly in dorsal flexion, and pain [6]. These alterations cause joint stiffness that causes a functional limitation that ultimately affects standing and walking in these patients [7]. The development of ankle arthropathy is related to lower tolerance of mechanical load during walking and standing. Accordingly, there are stability and walking alterations [8], which increase the risk of falls in these patients [9].

The term proprioception is applied to the sensory information that contributes to the sense of one’s own position, being an automatic and unconscious function. The joint mechanoreceptors do not exert any important reflex effect on the spinal motor neurons to control the periarticular muscles, and their action on the afferent fibers of the neuromuscular spindles is scarce [10]. Proprioceptive information is essential for keeping a stable posture and for the development of a normal gait [11]. This information travels to the central nervous system, but unlike other somatosensitivity-related components, much of the proprioceptive sense does not reach awareness. This is likely due to suppression as a consequence of motor signals [12] or inhibitions along the somatosensory pathways [13].

The interaction of the sensory, nervous, and musculoskeletal systems is responsible for the maintenance of postural stability [9]. The sensory inputs of joints and muscles are extremely important for the maintenance of orientation and postural balance.

In patients with hemophilia, the recurrence of hemarthrosis in the same joint impairs the function of mechanical receptors [14] causing pathophysiological modulation and deterioration of the musculoskeletal system, which affects balance in these patients from an early age [15].

The pain, typical of the development of hemophilic ankle arthropathy, is associated with a loss of strength, in addition to the generation of motor adaptations ranging from subtle compensations during the performance of tasks to the impossibility of performing pain-triggering movements or activities [16].

Therefore, biomechanical assessment and the development of joint, muscle, and functional assessment protocols are essential in the evaluation of patients with hemophilia. Magnetic resonance imaging (MRI) allows an accurate assessment of arthropathological changes. However, its high cost, the time required, and the need for sedation, especially in children, mean that it is not always a feasible option [17]. On the other hand, the development of a specific ultrasound protocol (Hemophilia Early Arthropathy Detection with Ultrasound; HEAD-US) has shown to be reliable in the evaluation of early inflammatory and destructive joint changes in the adult population with hemophilia [18], with the advantage of radiation-free safety and rapid assessment. Similarly, other measurement tools have been developed to measure joint damage secondary to hemarthrosis and the development of arthropathy in patients with hemophilia. The Hemophilia Joint Health Score is used to assess the joint status of the most impaired joints in these patients as a result of hemarthrosis: knees, ankles, and elbows [19]. This scale has shown high reliability (inter-observer coefficient = 0.83) [20], measuring 8 items (swelling, duration of swelling, muscle atrophy and strength, crepitus, mobility, and joint pain) with a score range of 0–20 points for each joint.

The hypothesis of our study is that patients with bilateral hemophilic ankle arthropathy have a poorer range of motion in ankle dorsal flexion, functionality, and muscle strength than subjects without ankle joint damage. However, observations in our daily clinical practice show that patients with advanced hemophilic arthropathy present good monopodal stability despite advanced joint damage, generalized hypotrophy, and chronic pain. The aim of this study was to analyze the differences in stability and balance, as well as in ankle dorsal flexion, functionality, and muscle strength, between patients with bilateral hemophilic ankle arthropathy and their healthy peers.

## 2. Materials and Methods

### 2.1. Design

A cross-sectional descriptive case–control study was performed. The guidelines for the reporting of observational studies described by the Strengthening The Reporting of Observational studies in Epidemiology (STROBE) were followed [21].

### 2.2. Ethical Considerations

The study was approved by the Medical Research Ethics Committee of the Virgen de la Arrixaca University Hospital of Murcia (ID code: 2022-7-2-HCUVA). All study participants were informed of the study objectives and signed the informed consent document prior to their inclusion. The study conforms to the Declaration of Helsinki for Human Experimentation. This study was registered, before starting the recruitment of subjects, in the international registry of clinical trials (www.ClinicalTrials.gov ID: NCT05425888).

### 2.3. Participants

Patients with hemophilia (cases) were recruited from two hemophilia patient associations in the regions of Andalusia and Murcia. The healthy participants (controls) were volunteers from the Murcia Region. Recruitment and data collection were carried out in November 2022.

The inclusion criteria of the cases were people (i) with a diagnosis of hemophilia, (ii) with severe phenotype of the disease, (iii) aged over 18 years, (iii) with a medical diagnosis of bilateral ankle arthropathy and clinical assessment of more than 4 points on the Hemophilia Joint Health Score [19], and (iv) with the ability to walk without technical aids. The controls had to meet the following criteria: (i) no history of ankle injury in the 12 months prior to the study; (ii) over 18 years of age; (iii) not sedentary in their activities of daily living; and (iv) not federated or competing in any sport.

All subjects had to sign the informed consent document to be able to take part in the study. Patients with other congenital coagulopathies, such as von Willebrand’s disease, were not included in the case group.

### 2.4. Sample Size

The sample size was calculated with version 17.0 of the STATA statistical package for Windows (Stata Corp LP, College Station, TX, USA). The sample size was based on the previous pilot study where the mean of the stability area was estimated in patients with hemophilia and bilateral arthropathy (6.21 mm^2^) with an accuracy of ±0.2 mm^2^, a 95% CI, and an amplitude of 0.6 mm^2^. The standard deviation of the stability area was SD = 0.654. The calculation was performed for amplitudes of 0.2, 0.4, 0.6, 0.8, 1.0, and 1.2 mm^2^. The sample size to estimate said mean with an accuracy of ±0.6 mm^2^ was 20 subjects.

### 2.5. Procedures

Patients with hemophilia (cases) were recruited from two Spanish patient associations. The main independent variables were collected with clinical and anthropometric data from patients with hemophilia. The healthy pairs (controls) were recruited among the teaching and research staff of the San Antonio Catholic University, located in the Region of Murcia (Spain). Homogeneity between the groups was achieved by age-quota matching of the case group and the healthy controls.

### 2.6. Outcome Measures

The main anthropometric data of the participants in the study (weight, height, and body mass index) and age were collected, as well as clinical data related to the joint condition of the case group, measured with the specific scale Hemophilia Joint Health Score [19]. This tool evaluates 8 items: inflammation and its duration, pain, muscle atrophy and strength, crepitus, and loss of flexion and extension. This instrument scores 0 to 20 points (maximum joint damage) per joint, with joint damage in both ankles and knees being evaluated (range 0–80):

- The primary variable was balance. The secondary variables were ankle dorsiflexion range of motion, functionality, and ankle dorsal strength. All evaluations were performed by a physiotherapist with more than 20 years of clinical experience. The measuring instruments used to measure the study variables were:
- Stabilometric analysis was performed with an Rs Scan^®^ pressure platform (RSscan Lab Ltd., Ipswich, UK) and a FootScan^®^ pressure measurement system (RSscan Lab Ltd., Ipswich, UK). This biomechanical examination device has shown moderate to good reliability. It measures foot pressure using an X-Y array of resistive pressure-sensitive sensors that are sequentially scanned. The system records the pressure data when the subject is standing or walking on the platform. The measurements were taken with the basic 0.5 m platform with 4096 sensors and resistive technology and 300 Hz data acquisition frequency. Static stability and balance were analyzed with eyes open and closed with a duration of 30 s [22].
- The Leg Motion^®^ device (Check your MOtion, Albacete, Spain) was used to evaluate ankle dorsal flexion. This tool has shown its high reliability and accessibility in measuring the range of motion of ankle dorsiflexion in adults. The test was performed according to weight-bearing lunge test procedures. The subjects stood on the Leg Motion system, with the first toe on the starting line and the knee in contact with the metal guide of the instrument. While the patients maintained that position, they were told how to lunge forward flexing the knee so as to touch the metal guide, which slides forward until reaching the maximum dorsal flexion of the ankle. During the test, patients were not allowed to take their heel off the ground, and the knee was to maintain contact with the metal guide at all times. Maximum dorsal flexion was defined as the maximum distance from the toe to the metal rod. Three measurements were taken on each ankle, with the average value being used for data analysis. All measurements were performed with the patient barefoot [23].
- The 2-Minute Walk Test (2MWT) was used to measure lower-limb functionality. The functional capacity to exercise was evaluated using this modified and validated version of the 6-minute format. This test was carried out in a closed corridor that was 30 meters long and delimited by cones. Before the test, the participants rested for at least 10 minutes [24]. They were asked to walk the circuit, going around the cones as fast as possible, but without running, for 2 minutes. Where necessary, they were allowed to slow down or stop to rest [25]. The distance covered, in meters, at the end of the 2 min was recorded by the evaluator [24].
- The maximum isometric force of the ankle flexor muscles in both legs was evaluated by dynamometry. A manual dynamometer was used, namely, Lafayette Manual Muscle Tester model 01165. This instrument has shown good intra- and inter-rater reliability. The unit of measurement of this instrument is Newtons per square centimeter (N/cm^2^), where the higher the score, the greater the muscle strength.

**Figure 1:**
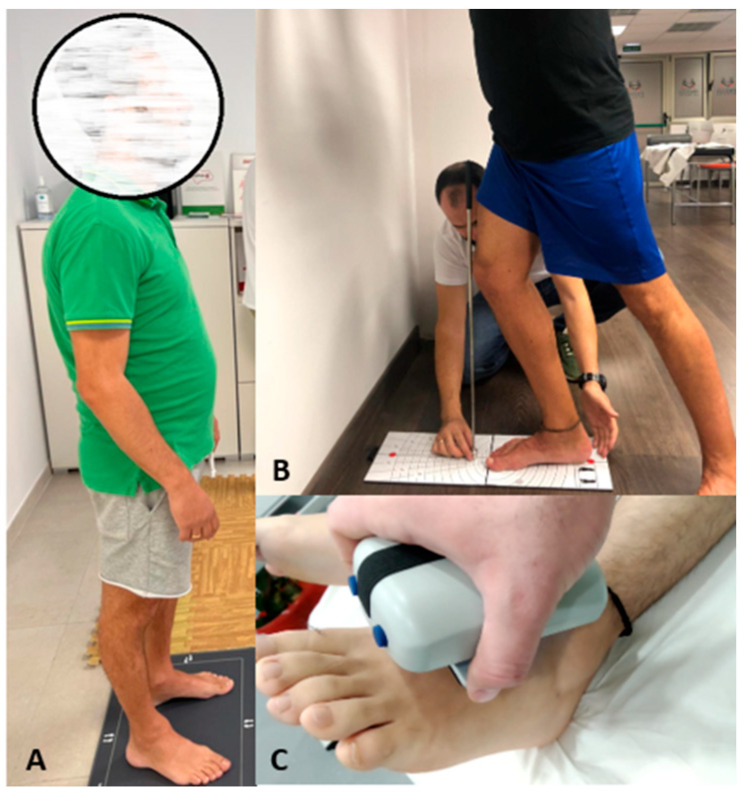
Assessments of the study variables. (**A**) Stabilometric analysis; (**B**) assessment with the Leg Motion device; (**C**) assessment of the maximum isometric strength of the ankle flexor muscles.

Figure 1 shows the assessments performed.

Prior to recruitment, a pilot study was conducted to calculate intra-observer reliability. The dependent variables were evaluated in seven patients with hemophilic ankle arthropathy, not included in the study.

### 2.7. Statistical Analysis

The SPSS 19.0 software (IBM SPSS Statistics for Windows; IBM Corp, New York, NY, USA) was used to perform the data analysis. Using the Shapiro–Wilk test, normality was assessed. A comparative analysis was made between the two groups by calculating the median and interquartile range of the study variables using the non-parametric Mann–Whitney U test. The differences in the Hodges–Lehman medians between the two study groups and the 95% confidence interval were described. The effect size of the comparisons between groups was determined by Cohen’s d, which was interpreted as very small (d < 0.20), small (d = 0.20–0.49), medium (d = 0.50–0.79), and large (d > 0.8) effect sizes [26]. According to the calculation parameters of the a priori sample size, the statistical significance was fixed at α < 0.05 for a 95% confidence interval (CI).

## 3. Results

Twenty-two participants were included in the study. Ten subjects had hemophilia (median age: 39.50; interquartile range [IR] = 16.75), with a median bilateral ankle joint damage score of 26.00 (IR: 10.00) points. The median age of the 12 healthy subjects was 39.00 (IR: 21.75) years. The descriptive characteristics of the two groups are shown in Table 1.

Excellent intra-observer reliability was noted in ankle dorsal flexion (ICC = 0.93) and lower-limb functionality (ICC = 0.98). High intra-observer reliability was observed in the variables muscle strength (ICC = 0.89), Maximum-X (ICC = 0.88), Maximum-Y (ICC = 0.84), and stability area (ICC = 0.83).

When comparing balance in the two groups, statistically significant differences were found in balance without visual support in the Max-Y variable (median difference [MD] = 2.83; Confidence Interval 95% [CI95%]: 0.33; 5.33; *p* = 0.04). Regarding the rest of the study variables, there were statistically significant differences (*p* < 0.001) in dorsal ankle flexion (MD = 17.25; CI95%: 15; 23), strength of the ankle flexor muscles (MD = 142.95; CI95%: 100.2; 177.3), and functionality (MD = 34.96; CI95%: 8.49; 61.43). Table 2 shows the statistics of central tendency and dispersion, the differences, and 95% confidence intervals in the variables between the two groups and the results of the effect size of the differences between patients with hemophilia and their healthy peers.

## 4. Discussion

Hemophilic arthropathy is characterized by the loss of joint mobility, the presence of chronic pain, muscle atrophy, and proprioceptive alterations. The present study analyzed the differences in stability and balance, as well as ankle dorsal flexion, functionality, and muscle strength, between patients with bilateral hemophilic ankle arthropathy and their healthy peers. There were no statistically significant differences between patients with hemophilia and their healthy peers in the main balance variables, with the exception of maximal anterior movement (*y*-axis) with eyes closed. However, there were statistically significant differences in the range of motion in dorsal flexion, muscle strength in dorsal flexion, and functionality.

In the analysis with visual support, in most of the stabilometric variables, there were no statistically significant changes between cases and controls. However, in the ellipse area variable (area of the calculated center of pressure of the ellipse), a high effect size was observed, which explains why patients with hemophilia present a smaller area of movement in their stabilometry than controls (3 mm^2^ vs. 6 mm^2^). On the other hand, in the stabilometric analysis with eyes closed, the variable that represents the maximum movement on the axis (anterior maximum displacement) presents a lower displacement in patients with hemophilia. Both findings could suggest that people with hemophilia have altered stability compared to healthy subjects.

We consider that joint damage is associated with a proprioceptive deficiency that favors less accuracy in postural control and more limited movements to avoid losing balance and possible falls. Joint involvement also implies damage to soft tissues and periarticular components; in this regard, we must pay attention to the upper and lower extensor retinacula and the lateral capsuloligamentous complex of the ankle, due to their important proprioceptive function [27]. There is some controversy regarding proprioceptive involvement after acute or chronic ankle injury [28] since there are studies reporting no differences between the groups of people with chronic ankle injuries or acute trauma and their healthy controls [29]. On the other hand, in patients undergoing total ankle arthroplasty, no differences in proprioception have been found between the operated and the non-operated side [30], although ankle arthroplasty presents poorer results in terms of proprioception and balance compared to total hip and knee arthroplasty [31].

Moreover, the main receptors sensitive to stretching and length changes are the muscle spindles, which are present in most skeletal muscles, being more abundant in muscles that require higher accuracy of movement. Motor innervation originates from static and dynamic myelinated γ-motor neurons that control the sensitivity of muscle spindle afferents as length detectors. On the other hand, the main stress-sensitive receptors are the Golgi tendon organs, located at the myotendinous junctions [32]. These sensory organs respond to changes in mechanical conditions: the length of the muscle (muscle spindles) or the active force (Golgi tendon organs), both being contraction receptors.

The presence of lower oscillations in patients with hemophilia may not relate to better stability [33]. Based on the joint damage in these patients, the results can be interpreted as a poorer ability of their joints to compensate for body movements [34] due to the decrease in strength and dorsal flexion [3]. Based on this, mechanical overexertion would occur that is not compensated by the musculoskeletal system [34].

In relation to pain, we need to consider the complex interaction of neurobiological and psychosocial factors that influence its perception; in this regard, in patients with hemophilic arthropathy, there could be unfavorable responses associated with hypervigilance, kinesiophobia, catastrophic thinking, and negative beliefs. Kinesiophobia, defined as an excessive, irrational, and debilitating fear of performing a movement, is the result of a feeling of vulnerability due to a painful injury and can be associated with pain and disability. Kinesiophobia alters the way people move, causing adjustments in motor behavior, which affect the performance of actions related to pain management and control [35]. Previous studies have detected that patients with hemophilia presented increased values of kinesiophobia [36]. High levels of kinesiophobia are common in subjects with chronic pain and are associated with sustained pain. In addition, negative correlations were noted between kinesiophobia and all elements of perceived quality of life except the emotional role. The development of musculoskeletal bleeding in patients with hemophilia causes intense pain that can justify the fear of suffering an injury and, therefore, limits their quality of life [36]. Accordingly, to avoid pain and protect the affected joints, patients with hemophilia may adopt more static and limited postures, thus reducing their movements in the stabilographic analysis.

The basis of most movements is due to balance control. If this control is affected, it likewise affects the development of basic activities of daily living. Our results found no statistically significant differences in most of the stabilometry variables despite the impact of hemophilic arthropathy. These results have been described previously by other authors [37]. In the daily lives of these patients, balance disorders reflect a disorder in the musculoskeletal system [38] that negatively impacts their quality of life and overall health condition.

Despite the advanced stage of joint damage of the population included in our study, the results are in line with other authors. The measurement of static balance does not usually show statistically significant differences in patients with hemophilia compared to their healthy peers. However, the measurement of dynamic balance does show poorer performance in patients with hemophilia [39]. In subsequent studies, the analysis of dynamic gait variables and dynamic stabilometry should be considered.

The advanced stage of joint deterioration in hemophilic arthropathy is characterized by the presence of muscle fibrosis, loss of joint space, alterations of joint structures, and pain [40]. This deformity thickens the posterior capsule of the ankle, causes the retraction of the Achilles tendon, and the collapse of the talus due to osteonecrosis and deterioration of the cartilage [41], which would justify the loss of dorsiflexor mobility, the decrease in strength [42], and consequently, the involvement of the biomechanics of gait (functionality of the patient).

### 4.1. Relevance to Clinical Practice

The findings presented in this study imply the need to recognize signs associated with impaired stability, decreased foot strength, or mobility in ankle dorsal flexion. These signs should be considered within a multimodal physiotherapy treatment program, which allows us to prevent complications and improve the clinical symptoms of patients with hemophilic arthropathy. The early diagnosis of these musculoskeletal disorders will help us to establish a musculoskeletal prophylactic approach to them. In this way, we will be able to offer patients with hemophilia greater independence and, with this, improve their perceived quality of life as much as possible.

Joint damage can affect joint range of motion, muscle strength, and the proprioceptive system, thus altering postural balance. However, although strength and balance, flexibility, mobilization, and aerobic exercises can produce a positive change in balance, more studies with a higher methodological quality are needed to evaluate the true efficacy of these techniques in improving balance in patients with hemophilia [43]. The development and generalization of new therapies with extended half-life clotting factors and bispecific monoclonal antibodies may facilitate the prevention of hemophilic arthropathy in younger patients. The absence of clinical hemarthrosis, the establishment of regular radiological follow-up protocols, and the implementation of adapted and controlled exercise programs for patients with hemophilia may lead to a generation of patients with hemophilia without joint damage or physical sequelae such as instability in the space of a few years.

### 4.2. Limitations of the Study

This study presents a series of limitations that should be considered. First, the small sample size should be taken into account when analyzing the results. Although the RSscan Footscan^®^ pressure platform 9 has shown good intra- and inter-rater reliability [44], it only measures forces perpendicular to the ground, disregarding forces in other planes. In addition, the data collected through the pressure platform are static. Therefore, we cannot simply infer that static positioning will directly affect dynamic movements. In future studies, the analysis of the dynamic variables of gait, dynamic stabilometry, and kinesiophobia should be considered.

## 5. Conclusions

Patients with bilateral hemophilic ankle arthropathy do not have statistically poorer stability and balance than their healthy peers. Patients with bilateral hemophilic ankle arthropathy present a reduced range of motion in ankle dorsal flexion. The ankle dorsiflexor force is lower in patients with hemophilia than in healthy subjects. When compared with subjects without ankle joint damage, patients with hemophilic arthropathy present poorer lower limb functionality.

## Figures and Tables

**Table 1:** Descriptive characteristics (mean and standard deviation) of the subjects included in the study in both groups.

Variables	Cases (n = 10)	Controls (n = 12)	Sig.
Age (years)	42.60 (16.30)	38.08 (10.27)	0.62
Weight (kg)	80.60 (17.35)	80.50 (8.70)	0.92
Height (m)	1.72 (0.11)	1.82 (0.08)	0.06
Body mass index (kg/m^2^)	27.07 (4.75)	24.34 (1.85)	0.18
Ankle joint damage (0–40)	23.80 (6.09)	-	
Knee joint damage (0–80)	18.10 (10.44)	-	

Ankle and knee joint damage: range of bilateral joint damage (20 points per joint) measured with the Hemophilia Joint Health Score; Sig.: Mann–Whitney U test for independent samples.

**Table 2:** Descriptive statistics of central tendency (mean) and dispersion (standard deviation) of the study variables in the two groups and intergroup differences.

Variables		Cases (n = 10)	Controls (n = 12)	MD (95%CI)	ES
Eyes open	Left (mm)	51.81 ± 8.63	49.22 ± 4.03	−2.58 (−8.40; 3.24)	−0.38
	Right (mm)	48.19 ± 8.64	50.49 ± 3.88	2.30 (−3.47; 8.08)	0.35
	Anterior (mm)	50.31 ± 8.35	46.24 ± 5.85	−4.06 (−10.39; 2.25)	−0.56
	Posterior (mm)	49.68 ± 8.36	53.75 ± 5.86	4.06 (−2.25; 10.39)	0.57
	Min-X (mm)	0.46 ± 2.15	0.61 ± 2.15	0.14 (−1.77; 2.06)	0.07
	Min-Y (mm)	−6.66 ± 2.79	−8.16 ± 3.55	−1.50 (−4.38; 1.38)	−0.46
	Max-X (mm)	4.53 ± 1.91	5.50 ± 1.72	0.96 (−0.65; 2.58)	0.53
	Max-Y (mm)	0.76 ± 2.29	1.24 ± 2.77	0.48 (−1.81; 2.77)	0.07
	Area (mm^2^)	3.80 ± 2.72	6.44 ± 3.42	2.64 (−0.14; 5.43)	0.83
Eyes closed	Left (mm)	49.52 ± 9.99	49.96 ± 3.84	0.44 (−6.06; 6.95)	0.06
	Right (mm)	47.14 ± 10.41	50.03 ± 3.84	2.88 (−3.84; 9.62)	0.37
	Anterior (mm)	48.39 ± 10.58	45.62 ± 5.75	−2.76 (−10.16; 4.63)	−0.32
	Posterior (mm)	48.27 ± 10.83	54.37 ± 5.75	6.09 (−1.42; 13.62)	0.72
	Min-X (mm)	1.63 ± 1.75	1.02 ± 2.41	−0.60 (−2.52; 1.31)	−0.34
	Min-Y (mm)	−7.90 ± 3.28	−7.58 ± 2.95	0.31 (−2.45; 3.09)	0.10
	Max-X (mm)	6.63 ± 2.48	5.83 ± 1.81	−0.80 (−2.71; 1.11)	−0.36
	Max-Y (mm)	0.23 ± 1.33	3.19 ± 3.66	2.96 (0.40; 5.51) *	0.67
	Area (mm^2^)	4.89 ± 3.35	6.83 ± 2.82	1.93 (−0.81; 4.68)	0.63
Dorsal flexion (degree)		−1.15 ± 6.74	14. 05 ± 5.87	16.98 (13.99; 19.96) **	7.46
Dorsal strength (N)		172.70 ± 58.77	309.50 ± 59.52	122.75 (95.55; 149.96) **	2.76
Functionality (m)		160.00 ± 57.5	195.00 ± 17.5	34.96 (8.49; 61.43) *	1.18

MD: mean difference; 95%CI: 95% confidence interval; ES: effect size; Min: minimum; Max: maximum. ** Corrected asymptotic significance of Yates continuity (*p* < 0.01). * Corrected asymptotic significance of Yates continuity (*p* < 0.05).

## Data Availability

The data that support the findings of this study are available on request from the corresponding author. The data are not publicly available due to privacy or ethical restrictions.
